# Forecasting Bifurcations from Large Perturbation Recoveries in Feedback Ecosystems

**DOI:** 10.1371/journal.pone.0137779

**Published:** 2015-09-10

**Authors:** Kiran D’Souza, Bogdan I. Epureanu, Mercedes Pascual

**Affiliations:** 1 Mechanical and Aerospace Engineering Department, The Ohio State University, Columbus, Ohio, United States of America; 2 Mechanical Engineering Department, University of Michigan, Ann Arbor, Michigan, United States of America; 3 Department of Ecology and Evolution, University of Chicago, Chicago, Illinois, United States of America; 4 Santa Fe Institute, Santa Fe, New Mexico, United States of America; University of Florida, UNITED STATES

## Abstract

Forecasting bifurcations such as critical transitions is an active research area of relevance to the management and preservation of ecological systems. In particular, anticipating the distance to critical transitions remains a challenge, together with predicting the state of the system after these transitions are breached. In this work, a new model-less method is presented that addresses both these issues based on monitoring recoveries from large perturbations. The approach uses data from recoveries of the system from at least two separate parameter values before the critical point, to predict both the bifurcation and the post-bifurcation dynamics. The proposed method is demonstrated, and its performance evaluated under different levels of measurement noise, with two ecological models that have been used extensively in previous studies of tipping points and alternative steady states. The first one considers the dynamics of vegetation under grazing; the second, those of macrophyte and phytoplankton in shallow lakes. Applications of the method to more complex situations are discussed together with the kinds of empirical data needed for its implementation.

## Introduction

Multiple stable dynamics coexist in ecological systems that have thresholds and breaking points. Transitions from one stable dynamics to another can often lead to drastic changes such as the extinction of species or the loss of ecosystems’ function [1, 2]. These changes are sometimes referred to as critical transitions in ecology to refer to catastrophic shifts. Dynamically, these changes represent bifurcations [3], that is, qualitative changes in temporal (or spatio-temporal) behavior caused by small changes in parameters. Specific types of bifurcations have different names in different fields, for instance fold or saddle-node bifurcations in mathematics are referred to as first order phase transitions in physics and as critical transitions in ecology. These bifurcations occur when two fixed points of the system eliminate each other when they come together, and results in a jump or discontinuity in the bifurcation diagram. The examples discussed in this work focus on such jump bifurcations. However, the methods are more general and work for continuous bifurcations such as transcritical bifurcations (referred to as second order phase transitions in physics) as well.

The bifurcation location in parameter space remains difficult to predict and current efforts seek to do so in the absence of a mechanistic model of the system [4]. Thus, the actual distance to such transitions, or in other words, how much further a parameter needs to change for the system to experience a significant qualitative change in its dynamics, remains an important empirical challenge; so does predicting the state of the system after this point is breached.

A number of indicators have been proposed to anticipate the approach to bifurcations based solely on empirical observations in the form of time series (see [5], for a review). In particular, a number of studies on ecological systems [6–9] have highlighted the presence of critical slowing down [3] (CSD) in measurements leading up to a breaking point. Model-less methods [8, 10, 11] based on CSD have been used to show that a bifurcation is being approached in a number of systems in nature. These CSD-based methods rely on indicators such as an increased autocorrelation [12, 13] and variance [14, 15] obtained from measurements on the system’s recovery from *small* perturbations. More recently, the phenomenon known as ‘flickering’ has been proposed to detect coexisting equilibria and their associated basins of attraction, by taking advantage of process noise [15–17]. The need to quantify the uncertainty associated with predicting bifurcations has also been emphasized including in a recent approach that estimates the location of the breaking point and the likelihood of a false alarm or failed detection for a transition involving alternative equilibria [4, 18]. Controlled experiments with the cladoceran zooplankton *Daphnia magna* have also lent support to the usefulness of leading indicators [10].

**Fig 1:**
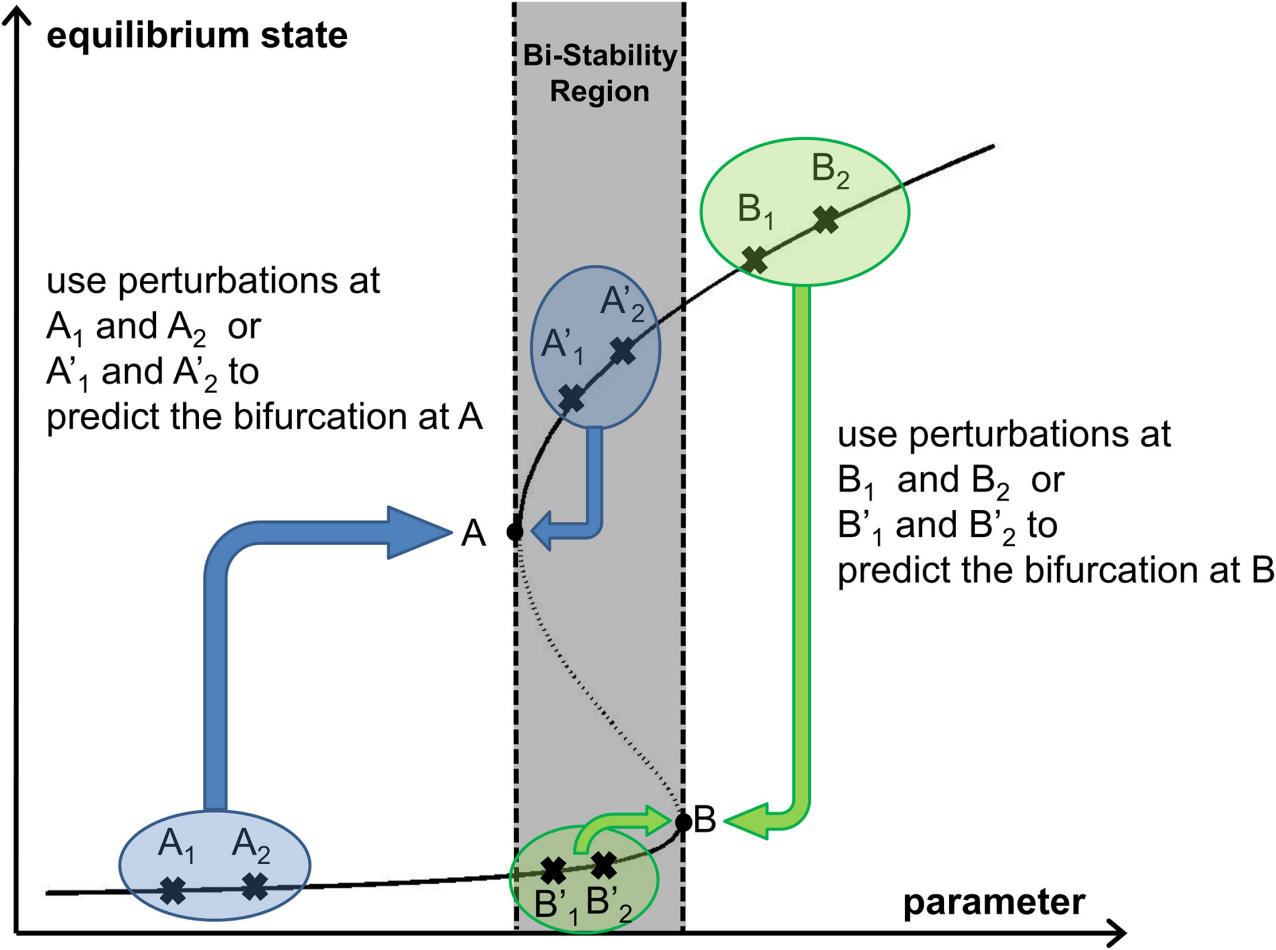
Bifurcations are predicted using data from the pre-bifurcation regime. The method predicts the edges of the bi-stable region using data either from outside or inside of this zone. In this example, the bifurcation at *A* is predicted from data collected at *A*_1_ and *A*_2_ (or $A_{1}^{'}$ and $A_{2}^{'}$), while the bifurcation at *B* is predicted from data at points B_1_ and B_2_ (or $B_{1}^{'}$ and $B_{2}^{'}$).

In this work, we present a complementary approach to predict the location of bifurcations such as breaking points without an actual model, that is also able to forecast the behavior of the system after the bifurcation. This capability provides a means to assess from data on transient recoveries, not only the kind of bifurcation a system is approaching, but also whether this transition involves a bi-stable region in which alternative equilibria coexist and are locally stable. The approach is qualified as model-less because it is not based on a mechanistic set of equations representing the underlying processes involved in the system dynamics. Because the proposed method makes use of transient responses to *large* perturbations, it applies in advance of the bifurcation, and for the specific case of alternative steady-states, well in advance of entering the bi-stable zone. In this case, the approach provides a prediction of the distance to the bifurcation that marks the entrance to the bi-stable regime and precedes a tipping point (Fig 1, see also Figure 2 in [19]). The method also applies however within the bi-stable zone, for smaller perturbations, complementing existing approaches that are based on return trajectories from sufficiently small distances to equilibria in state space [19].

By contrast, recoveries from large perturbations are exploited in the proposed approach. These perturbations can arise from natural events or human interference. It is the influence of ‘ghost’ attractors, as reflected through changes in the recovery rates, that provides the basis to extrapolate from at least two such trajectories the location and type of bifurcation. When a single recovery is available, insight can be gained into the expected type of bifurcation, but no quantitative predictions can be made with this limited amount of information.

We demonstrate here the proposed method with simulation data generated with two established ecological models whose bi-stable dynamics have been studied extensively: a vegetation grazing ecosystem [1, 6, 9, 20, 21] and a feedback system between macrophytes and phytoplankton in shallow lakes [19, 22–24]. We further examine the robustness of the approach to different levels of measurement noise. We focus on bi-stable systems because of their relevance to tipping points, not just in ecology but in a wide variety of fields concerning the dynamics of climate [11], neurons [25], global finances [26], aeroelasticity of aircraft [27], power grids [28], and other biological interactions (such as those underlying the onset of epileptic seizures [29], asthma attacks [30] and infectious disease transmission [31]). We further discuss that the proposed method applies to a broader class of dynamics that exhibit CSD while undergoing a variety of bifurcations.

## Methods

### Method

**Fig 2:**
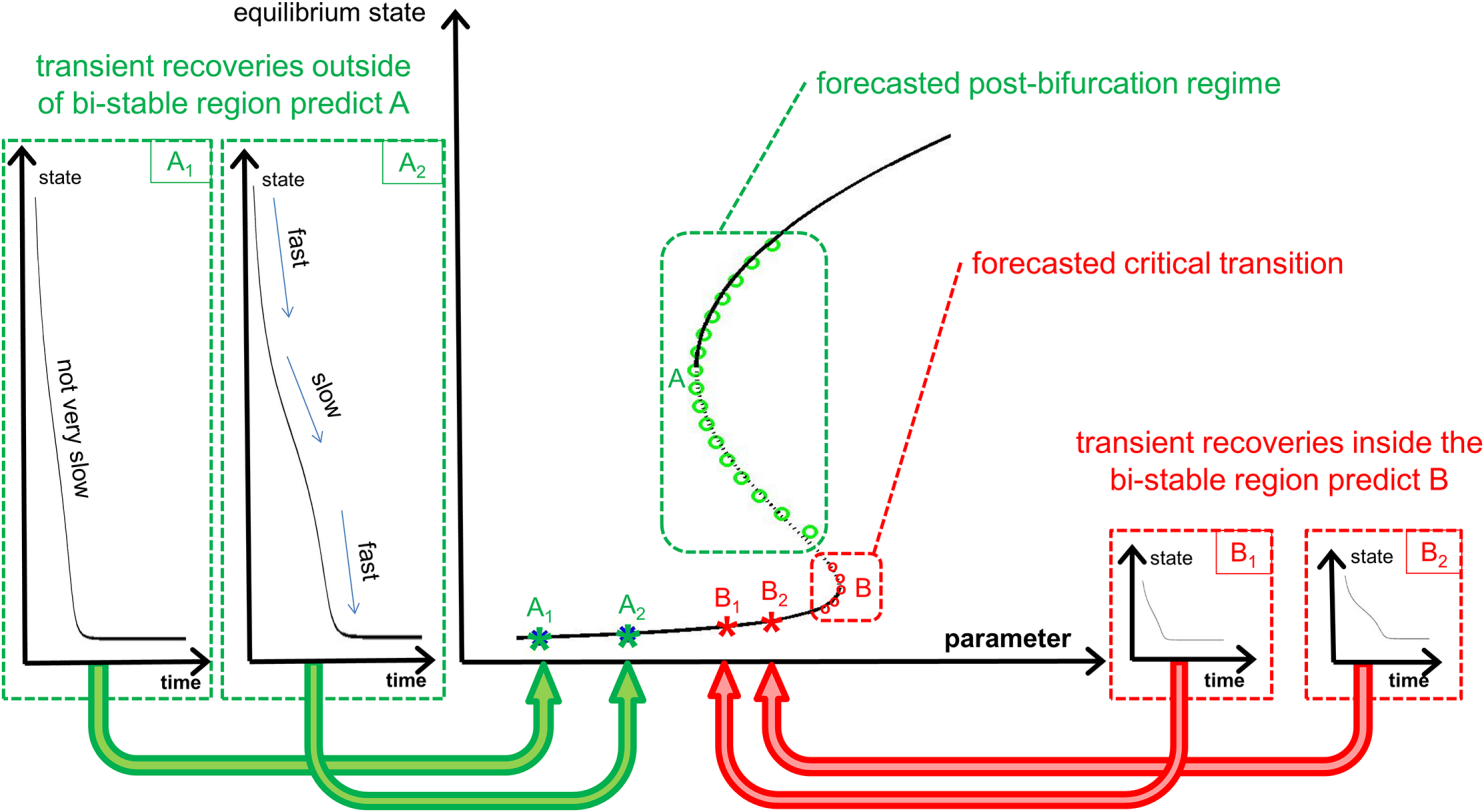
Overview of the method to forecast bifurcations using recoveries from perturbations. The bifurcation diagram with both the stable (solid line) and unstable (dotted line) branches of the system dynamics are shown. The methodology predicts the post-bifurcation regime (A) using transient recoveries from perturbations from two locations outside the bi-stable region (A1 and A2). It can also predict the bifurcation (B) using transient recoveries within the bi-stable region (B1 and B2). The recovery trajectories are shown in the four insets. The areas where slowing down is observed align with the value of the equilibrium state of the bifurcation point. The method takes advantage of the changes in the recovery rates, and of the CSD generated by the presence of ‘ghost’ equilibrium, to extract information on the location and kind of bifurcation.

The general concepts and the basis of the technique are summarized in Fig 2. The types of transient recoveries needed at two parameter values to forecast the bifurcation at *A* and bifurcation at *B* are shown in boxes *A*_1_ and *A*_2_, and *B*_1_ and *B*_2_, respectively. Note that the dynamics slow down as the state of the system (but not the parameter which is fixed over the course of the transient recovery) approaches the bifurcation. The region where the slow down is most pronounced corresponds to the equilibrium state of the nearest bifurcation with respect to the critical parameter. This is the fundamental idea that is exploited by the method to forecast the bifurcation and post-bifurcation regime.

The idea is to relate local reductions in the rate of recovery (to an equilibrium) to the presence of another fixed point nearby in the parameter space. Note that this does not refer to the closest fixed point in the physical coordinates, or state space, of the system but to the closest fixed point in the parameter space. The local slowing down is due to a weak ghost node phenomenon where the presence of this other, unknown, fixed point affects the recovery rate of the transient recovery [3]. The states of the system where this recovery has the slowest rate are closest (in the parameter space) to a fixed point. For example, the amplitude/state marked as ‘slow’ in the recovery *A*_2_ in Fig 2 corresponds to the amplitude/state *A* shown in the bifurcation diagram in Fig 2. The slow recovery at that amplitude/state is closest to point *A* in the parameter space (i.e. the horizontal coordinate in the bifurcation diagram). Also, the method uses the observation that in general the faster the recovery, the farther the nearest fixed point in parameter space.

Consider a system whose dynamics contain several manifolds. Specifically, as a parameter *μ* varies, the system is such that its dynamics in one of the manifolds exhibit a bifurcation at a critical parameter value *μ*_*c*_, and that the manifold is one-dimensional. Thus, the dynamics in that manifold are characterized by a (physical, measurable) coordinate *x*, and a governing equation given by

$$\begin{array}{c} \frac{dx}{dt}=h\left(x,\mu \right). \end{array}$$ (1)

The function *h*(*x*, *μ*) is not known but is required to be smooth with respect to *μ* so that its Taylor series with respect to this parameter near its critical value *μ*_*c*_ can be written as

$$\begin{array}{c} h\left(x,\mu \right)\approx h\left(x,\mu_{c}\right)+\left(\mu -\mu_{c}\right)\frac{dh(x,\mu_{c})}{d\mu }+\text{HOT}. \end{array}$$ (2)

Note that this implies neither that the system dynamics have been linearized near an equilibrium, as typically done when considering Jacobian matrices for the purposes of local stability analysis, nor that the dynamics are of low amplitude. Importantly, the above expansion is not relative to the state of the system, for example, close to the equilibrium; rather, the linearization is only in the parameter space about the bifurcation parameter *μ*. Thus, neglecting the higher order terms (HOT) provides an approximation of increasing accuracy as the system approaches the bifurcation. If the application demands it, however, the HOT can be accounted for, as presented in the Discussion section. For the models discussed herein, Eq (2) provides excellent accuracy, and the HOT are identically zero (because the models can be cast in a form where these terms are effectively zero). In other cases, where the HOT are not identically zero, they are typically small sufficiently close to *μ*_*c*_.

For clarity of notation, denote $g(x)=1/\frac{dh(x,\mu_{c})}{d\mu }$ and $f(x)=h(x,\mu_{c})/\frac{dh(x,\mu_{c})}{d\mu }$, with the functions *f*(*x*) and *g*(*x*) generally unknown. From Eq (2), the governing equation for the system in the inertial manifold where the bifurcation takes place can be written as

$$\begin{array}{c} g\left(x\right)\frac{dx}{dt}=\left(\mu -\mu_{c}\right)+f\left(x\right). \end{array}$$ (3)

The key to predicting the post-bifurcation dynamics is to use measurements of the dynamics due to perturbations in the pre-bifurcation regime to estimate $\eta (x,\mu )=g(x)\frac{dx}{dt}$. Specifically, Eq (3) shows that

$$\begin{array}{c} \eta \equiv g\left(x\right)\frac{dx}{dt}=\left(\mu -\mu_{c}\right)+f\left(x\right). \end{array}$$

Hence, *η* varies linearly with *μ* (when HOT in Eq (2) are negligible).

To estimate *η* from measurements of *x* over time, consider two time series of measurements (denoted by *x*_1_(*t*_1_) and *x*_2_(*t*_2_)) of the ecosystem response for two different values of *μ* (denoted by *μ*_1_ and *μ*_2_). The time series are also used to estimate $\frac{dx_{1}}{dt}$ and $\frac{dx_{2}}{dt}$ (e.g., using finite differences). Now if one chooses points where *x*_1_(*t*_1_) = *x*_2_(*t*_2_) = *x*, then Eq (3) yields

$$\begin{array}{c} g\left(x\right)\frac{dx_{1}}{dt}=\left(\mu_{1}-\mu_{c}\right)+f\left(x\right),\\ g\left(x\right)\frac{dx_{2}}{dt}=\left(\mu_{2}-\mu_{c}\right)+f\left(x\right). \end{array}$$

Subtracting these relations yields

$$\begin{array}{c} g\left(x\right)=\frac{\Delta \mu }{\Delta v},. \end{array}$$

where Δ*μ* = *μ*_2_ − *μ*_1_, and $\Delta v=\frac{dx_{2}}{dt}-\frac{dx_{1}}{dt}$. Hence, *η* becomes

$$\begin{array}{c} \eta =\frac{\Delta \mu }{\Delta v}\frac{dx}{dt}. \end{array}$$ (4)

**Fig 3:**
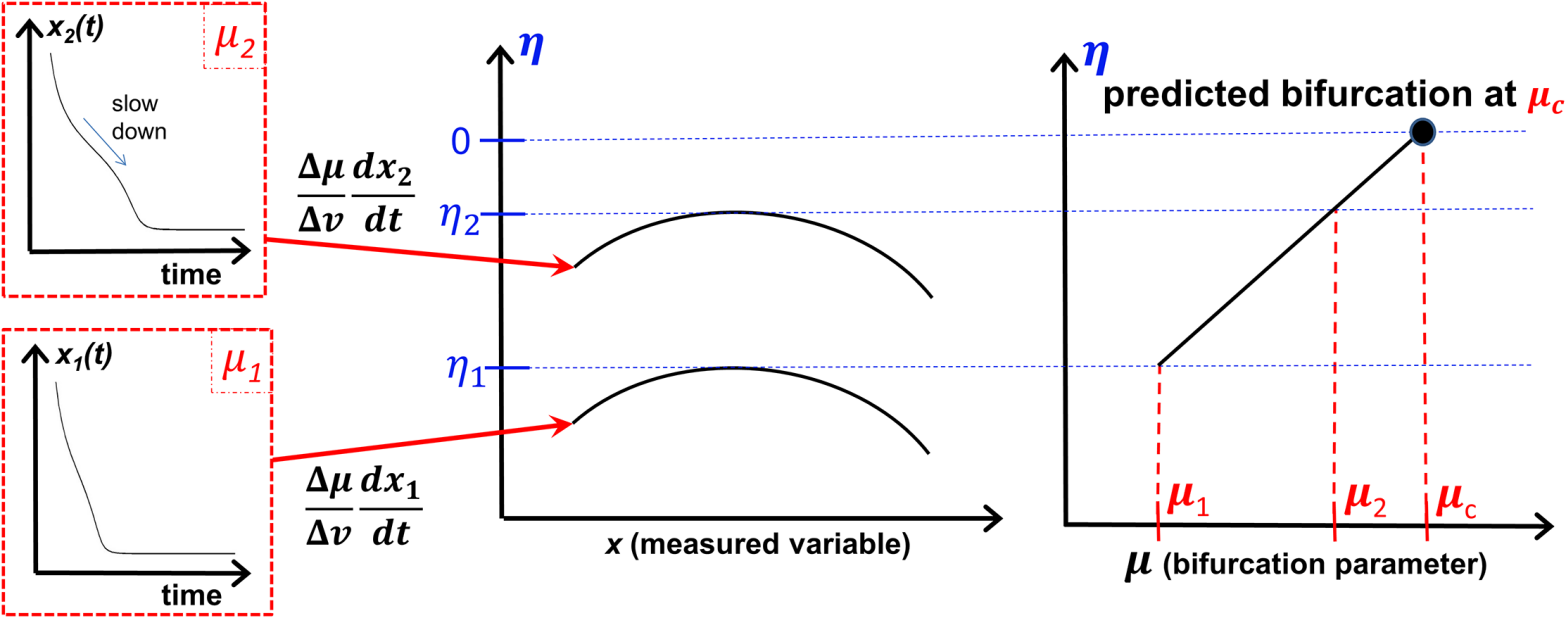
Description of the method to forecast the bifurcation point *μ*_c_ using recoveries at two bifurcation parameter values (*μ*_1_ and *μ*_2_). Using Eq (4), *η* curves are generated for each recovery, and their corresponding peaks are used to predict the bifurcation point *μ*_c_. Additional points along the *η* curves can also be used to predict the amplitude in the post bifurcation regime.

An overview of how the method is used to predict the bifurcation point is shown in Fig 3. Response measurements from at least two recoveries at distinct bifurcation parameter values are measured. From these measurements, the *η* curves are computed and the bifurcation parameter is obtained. Note that additional points (outside of the peak points along the *η* curves) can be used to compute the amplitude in the post bifurcation regime.

### Context and Assumptions

The proposed method is designed for systems which exhibit critical slowing down (CSD). Here, we demonstrate the technique with two ecological models that have bi-stability and hence S-shaped bifurcation curves (Fig 1). The method relies on observations of how a system recovers from perturbations at two or more conditions (before a bifurcation, occurs). Distinct from other existing methods, the method can predict the edges of the bi-stable zone without entering it, as shown in Fig 1. The bifurcation point *A* (or *B*) in Fig 1 is forecasted from measurements collected when the critical parameter is at *A*_1_ and *A*_2_ (or symmetrically, at *B*_1_ and *B*_2_). Hence, the bifurcation can be predicted from measurements of recoveries from perturbations outside the region of bi-stability. This would indicate the entrance into a region of coexisting equilibria, which could then lead to a tipping point. One could then direct attention to the danger posed by perturbations shifting the system between basins of attraction, and rely on other, existing, complementary methods based on small perturbations. Additionally, the method can also predict the bifurcation at *A* (or *B*) using measurements from within the bi-stable region using in this case, measurements at points $A_{1}^{\prime }$ and $A_{2}^{\prime }$ (or $B_{1}^{\prime }$ and $B_{2}^{\prime }$). Thus, the approach effectively predicts the nearest bifurcation point.

When forecasting a subcritical bifurcation from outside the bi-stable region, perturbations larger than the critical point are considered large, i.e. the perturbed states can be larger than point *A* in Fig 1. When forecasting from inside the bi-stable region, perturbations can be as large as possible as long as they do not cause the system to jump to the alternative states. When forecasting a supercritical bifurcation, the size of the perturbations has to be at least as large as the largest state of the forecasted bifurcation diagram. Because large perturbations inside a bi-stable region are susceptible to causing jumps in the system dynamics, they can be desirable or not depending on the application. For example, in the case of infectious disease elimination, it is desirable to transition from the upper branch to the lower one or disease-extinction state. By contrast, when predicting emergence, the drastic change is in the opposite direction and is not desirable. This difference must be taken into account when the recovery trajectories arise from planned intervention rather than from their opportunistic sampling, especially because one does not know *a priori* the current state of the system relative to that of a potential transition or bi-stable region.

Nevertheless the transient trajectory in the recovery from large perturbations provides a unique ability to forecast what the behavior of the system will be after a bifurcation without the system actually passing a breaking point or entering the bi-stability region. The size of the perturbation is related to the specific application and the location of the fixed point of the system. Consider the responses shown in Fig 2. The perturbations used for this method, if measurements were taken at *B*_1_ and *B*_2_, have to be relatively small, otherwise the system could switch to the alternative stable state at these points (and not recover to the same state where it started from). In contrast, arbitrarily large perturbations (in terms of the amplitude of the dynamics) can be used for this method if measurements are taken at *A*_1_ and *A*_2_, since there are no other fixed points at these parameter values. All of the recovery back to the equilibrium state might not be useful if the perturbation is very large, but the portion of the recovery where the dynamics slow down (see the plot of the transient recovery at *A*_2_ in Fig 2 where labelled as “slow”) due to the bifurcation at a different parameter value is the information used in the method.

Note that applied within the bi-stability region, the method does not require relatively large perturbations to predict the bifurcation. The perturbations only have to take the state of the system past the equilibrium of the bifurcation. Also, note that the method cannot be applied if the system is alternating between two stable states or flickering. Flickering prohibits the system to consistently recover to the same state after perturbations which is a requirement of the method. Hence, without further development, the method cannot be directly applied to systems with flickering.

In addition, the forecasting method does not rely on a model of the processes governing the dynamics of the system, although it requires knowledge of the bifurcation parameter whose distance to the critical point is the focus of the analysis. The approach has similarities with Lim and Epureanu [32, 33] where tipping points in the dynamics of electro-mechanical systems (e.g., atomic force microscopes) were studied. We later discuss both this requirement and the differences with this previous work.

There are a few key assumptions used in this forecasting approach. The first is that the bifurcation parameter *μ* is sufficiently close to its critical value *μ*_*c*_ for the dynamics to experience a slow down. This is an assumption made by all forecasting methods that use CSD phenomena [8, 10–15].

**Fig 4:**
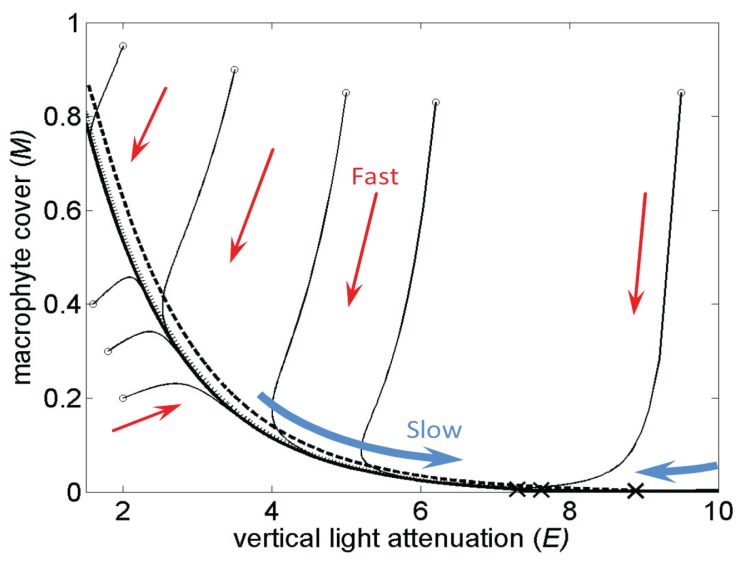
The inertial manifold (i.e., the invariant set in which the dynamics is slowest in time) is low dimensional and varies slowly with the bifurcation parameter.

The second key assumption used in this method is that the inertial manifold (the invariant set in which the dynamics is slowest in time, for example see Fig 4) is one dimensional, as is the case in many bifurcations observed experimentally. The dynamics of a system typically contain sets of states which are invariant; namely if the system starts from any state in a set, it remains in that set at all times. The slowest manifold is sometimes referred to as the inertial manifold [34].

A third assumption is that the dynamics varies smoothly with the parameter. A fourth assumption is that the inertial manifold of the dynamics varies smoothly with the parameter, as discussed in the next section.

Although, in this work, the method is demonstrated on saddle node bifurcations, the assumptions hold true for a larger class of bifurcations. For example, transcritical bifurcations can be forecasted by the proposed method. However, the requirements do not hold for other bifurcations such as period doubling.

### Discussion of the Method

Typically, the inertial manifold of the dynamics is low dimensional near the bifurcation even though the system may be high dimensional. Hence, as few as a single variable of the system has to be measured in order to apply the method. This variable can be chosen arbitrarily as long as it is not in a space nearly orthogonal to the inertial manifold. Because most of the information about the bifurcation is in the inertial manifold, the measured variable should be able to reflect some of that information. Also, forecasting requires the inertial manifold to vary slowly with the bifurcation parameter.

The computation of *η* does not require a model of the ecological system, and therefore, explicit knowledge of the functions *f* or *g*. It requires instead measurements of the coordinate *x* of the system along its transient trajectory in the recovery from perturbations from at least two values of the parameter *μ*. Note that the variable *η* differs substantially from the variable *λ* introduced by Lim and Epureanu [32]. The variable *λ* is in essence $\frac{\dot{x}}{x}$ and can only be used for a much smaller class of bifurcations as it assumes a more particular form of the underlying model of the system.

Since measurement errors are unavoidable, a filter for the resulting noise in *η* is obtained by applying a curve fit through the data obtained using Eq (4). This fit for *η* is linear in *μ* and polynomial in *x*. The values of *η* are all from the pre-bifurcation regime, where *η* is negative because the dynamics exhibit a stable recovery (see Fig 3). The bifurcation occurs when *η*(*x*, *μ* = *μ*_*c*_) is at its peak (largest value) where the curve approaches zero. The fixed points in the post-bifurcation regime can be extracted from the rest of the *η* curve. The method can use noisy observations from different perturbation levels at the same conditions (i.e., same bifurcation parameter value). In those cases, averaging can be applied to mitigate the effects of noise when observations of recoveries from several perturbations at the same *μ* value are available.

Depending on the amount and type of data available additional noise filtering techniques can be applied to eliminate noise from the transient recoveries.

### Overview of the Method

The method can be summarized as the following steps:

1. Measure at least one state *x* of the system’s response as it recovers from perturbations (that are natural or induced) from at least two parameter (*μ*) values. (If perturbations from only one *μ* value are available or if *μ* is unknown, then the method can be used to predict the expected type of bifurcation, but cannot precisely predict the location of the bifurcation.)
2. Apply noise filtering techniques to available transient recoveries. For example, when multiple recoveries are available at the same parameter value, then these can be first shifted in time (allowed because the system is time-invariant) so that they have an intersection which is then considered a common starting point. Starting from that point, the recoveries can be averaged (see applications below).
3. Compute *η* using Eq (4) for each *μ* value.
4. Fit a curve through the computed *η* values for each *μ* value. If data from only one *μ* value is available, then the *η* curve can be interpreted qualitatively based on its shape (which indicates the type of post-bifurcation dynamics) and on how close the peak of the curve is to zero (which relates to the proximity of the bifurcation point).
5. Finally, find the peaks of each *η* curve and extrapolate them to find the *μ* value where the bifurcation occurs. In a similar manner, choose points on each side of the peak of the *η* curve to predict the post-bifurcation dynamics (i.e. the branches of the bifurcation diagram). This linear extrapolation is exact when the HOT in Eq (2) are zero.

### Two Examples from Ecological Systems

To illustrate and evaluate the method under measurement noise, ‘data’ are generated from simulation of two ecological models. The first is for the dynamics of vegetation under grazing [1, 21] characterized by

$$\begin{array}{c} \frac{dV}{dt}=rV\left(1-\frac{V}{k}\right)+\frac{cV^{2}}{V^{2}+h^{2}}, \end{array}$$

where *V* is the vegetation biomass, *r* is the logistic growth rate, *k* is the carrying capacity, *c* is the bifurcation parameter (which is the opposite of the grazing rate), and *h* relates to the grazers’ intake capacity. Recent studies of this model address the effects of measurement noise and time delays on its dynamics [9], the recovery rate as a qualitative indicator of a dynamical shift [19], as well as evidence for spatial correlations providing better early warnings than temporal indicators from time series [6].

This ecological system has an S-shaped bifurcation curve (Fig 2), and its model can be cast in the form of Eq (3) using

$$\begin{array}{ccc} f(V) & = & r\left(V^{2}+h^{2}\right)\left(\frac{1}{V}-\frac{1}{k}\right),\\ g(V) & = & \frac{V^{2}+h^{2}}{V^{2}}, \end{array}$$

although these equations are not needed. The analytical forms of f(V) and g(V) are never used; rather an estimate of *η* is calculated using Eq (4) and only measurements of *V*.

As a second demonstration, forecasting is applied to the nonlinear dynamics created by the feedback between macrophytes and phytoplankton in a lake [19, 22–24]. This system is more complex than the vegetation grazing one, and its behavior can be described by two coupled differential equations. The ecosystem dynamics are governed by

$$\begin{array}{ccc} \frac{dE}{dt} & = & r_{E}E\left(1+E\frac{h_{M}+M}{eh_{M}}\right),\\ \frac{dM}{dt} & = & r_{M}M\left(1-M\frac{h_{E}^{4}+E^{4}}{h_{E}^{4}}\right), \end{array}$$

where *M* is the macrophyte cover, *E* is the vertical light attenuation, *r*_*E*_ and *r*_*M*_ are growth rates, *h*_*M*_ is the critical macrophyte cover, *h*_*E*_ is the critical light attenuation for the vegetation, and *e* is the (monitored) bifurcation parameter.

The bifurcation diagrams (one for *E* and one for *M*) are S-shaped, and the inertial manifold of the dynamics is one dimensional. This manifold is shown in Fig 4 for three different values of parameter *e* (−7.5: thick solid line, −7.8: dotted line and −9: dashed line) together with eight trajectories of the system (thin solid lines) for *e* = −7.5. The manifold varies slowly with *e* (e.g., the manifolds for *e* = −7.5 and *e* = −7.8 almost overlap). The inertial manifold is approximately tangent to the *E* axis near the fixed point (denoted by one × symbol for each *e* value). Thus, *E* is the preferred variable to measure. Because the tangency is not perfect, there is some variation in *M* as the fixed point is approached. Hence, *M* is not in a space nearly orthogonal to the inertial manifold and could also be used, but the results would be more sensitive to noise. Note that the analytical forms of functions *f*(*E*) and *g*(*E*) as well as *f*(*M*) and *g*(*M*) could be computed for this model, but they are not needed for the forecasting method.

## Results

We demonstrate next the method computationally to examine its application to data collected from a system’s dynamics, as typically done to investigate and evaluate analyses based on indicators [19]. Thus, the models for a vegetation grazing ecosystem and the feedback between macrophytes and phytoplankton in a lake are used to obtain simulated data. The parameters of the former are given in Table 1 and those for the latter in Table 2. Random errors were applied to the simulated data to introduce measurement noise at specified levels. Results below demonstrate that the forecasted bifurcation and the state of the system after this transition match the actual behavior.

**Table 1:** Parameter values of the vegetation grazing model used to produce simulated measurement data.

Parameter	Value	Physical Meaning
*r*	1/day	Growth rate for the population
*k*	5	Carrying capacity (units of vegetation)
*h*	0.5	Animal intake capacity

**Table 2:** Parameter values of the model of the feedback between macrophytes and phytoplankton in a lake used to produce simulated measurement data.

Parameter	Value	Physical Meaning
*r*_*E*_	0.1/day	Growth rate
*r*_*M*_	0.05/day	Growth rate
*h*_*E*_	2/m	Critical light attenuation to get vegetation
*h*_*M*_	0.2	Critical macrophyte cover

**Fig 5:**
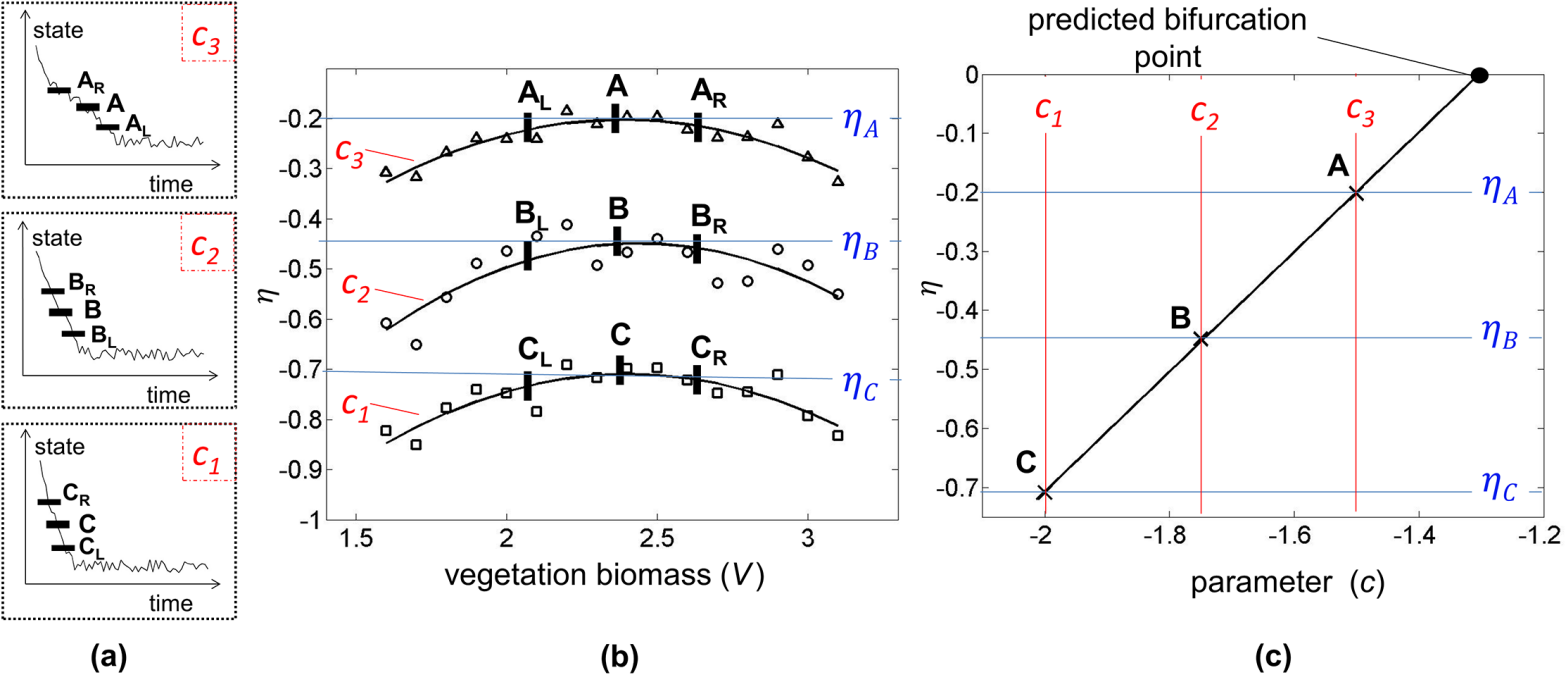
Analysis of recovery from perturbations to forecast a bifurcation. (a) Noisy observations at three conditions (i.e., three bifurcation parameter values) for a vegetation grazing ecosystem recovering from perturbations. Labels *A*, *B*, and *C* indicate regions where the dynamics slows down. The plot for region A corresponds to the parameter value closest to the bifurcation, and the plot for region *C* corresponds to the parameter value farthest from the bifurcation. (b) A variable *η* is obtained from the transient response data near the slowing down region and plotted versus the vegetation biomass. Curves are fit for each condition. Note that labels *A*, *B*, and *C* correspond to the peaks of each curve. Additional points are indicated to the left (subscript *L*) and right (subscript *R*) of the peaks on each curve. (c) One *η* value obtained from each curve (*A*, *B* and *C*) is plotted versus the bifurcation parameter *c*. The location of the bifurcation is forecasted by approximating where the line connecting these points intersects the *x*-axis. The post-critical regime can be forecasted in a similar manner by approximating where lines defined by other sets of points such as *A*_*L*_, *B*_*L*_ and *C*_*L*_, and *A*_*R*_, *B*_*R*_ and *C*_*R*_ cross the *x*-axis.

Examples of recovery at three different grazing values (*c*_1_, *c*_2_ and *c*_3_) for the vegetation grazing ecosystem are shown in Fig 5(a), where 30% random errors (with zero mean) were added to the observation data. The value of $\eta =\frac{\Delta c}{\Delta \frac{dV}{dt}}\frac{dV}{dt}$ is extracted from these observations, with a central differencing scheme used to estimate $\frac{dV}{dt}$ from measurements of *V* over time. To obtain *η*, data from at least two values of *c* are used, based on the observed recoveries (Fig 5(b)).

Observations in the regions of slowing down (near labels *A*, *B* and *C* in Fig 5(a)) are used to estimate *η* because they are the least sensitive to errors/noise and they provide insight into the post-bifurcation regime. Data very close to, or very far from, the steady state response are not used; this is because the former are more profoundly affected by noise while the latter, far from the steady state, can be off the inertial manifold. Once a perturbation occurs, the state of the system is not necessarily on the inertial manifold, but soon afterwards the dynamics will move onto the inertial manifold (since convergence to this manifold is fast, e.g. Fig 4).

To filter out much of the noise, a polynomial curve is fitted through each data set for the three grazing values (*c*_1_, *c*_2_ and *c*_3_). The peak values of *η* (*η*_*A*_, *η*_*B*_ and *η*_*C*_) denoted by the labels *A*, *B* and *C* on the curves in Fig 5(b) are used to forecast the value of *c* where the bifurcation takes place, as shown in Fig 5(c). In a similar manner, the post-bifurcation regime is forecasted by points such as *A*_*L*_, *B*_*L*_ and *C*_*L*_, and *A*_*R*_, *B*_*R*_ and *C*_*R*_. Note that the linearity seen in Fig 5(c) is due to the negligible HOT in Eq (2) and a linearization is not being done. This is true for many systems and becomes more accurate as the bifurcation is being approached. If the HOT were not negligible then the points would not form a straight line, and a higher order fit of *η* vs. parameter would be needed. For such a higher order fit, recoveries at three or more parameter values would be necessary.

**Fig 6:**
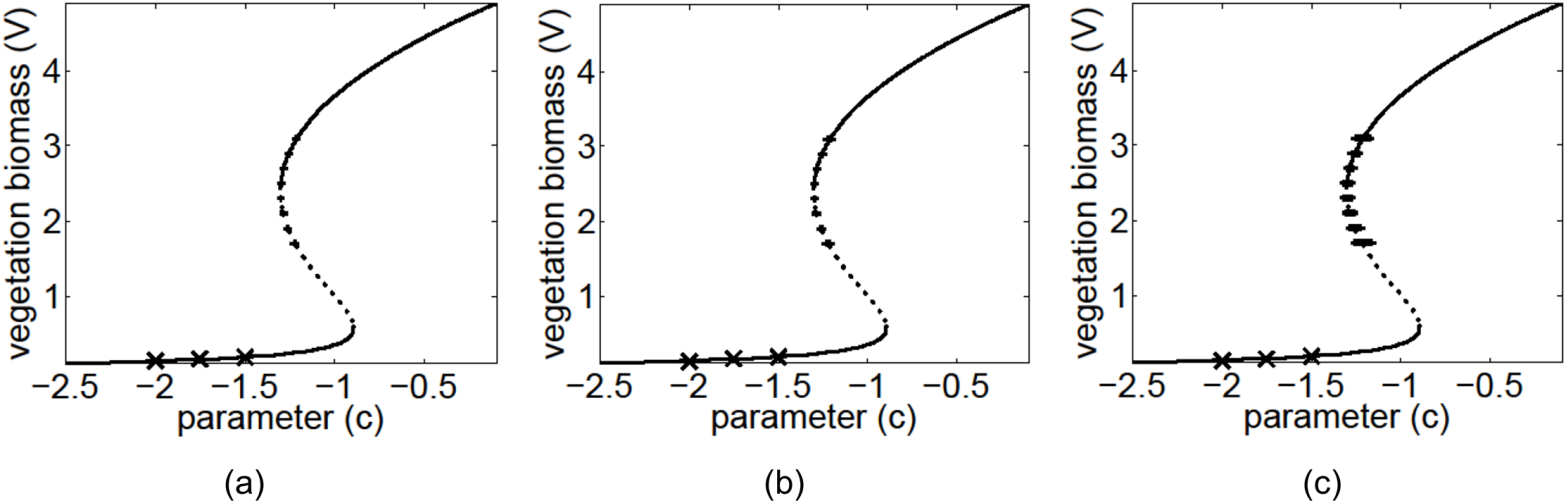
Predicted bifurcation diagrams for the vegetation model using 100 recoveries at three pre-bifurcation conditions. The exact bifurcation diagram with both the stable (solid line) and unstable (dashed line) branches are shown in each plot. Transient data were collected at three locations (indicated by labels X) in the pre-bifurcation regime. The predicted post-bifurcation regime is shown together with the standard deviation error bars for 100 separate noisy realizations for (a) 10% noise, (b) 20% noise, and (c) 50% noise.

**Fig 7:**
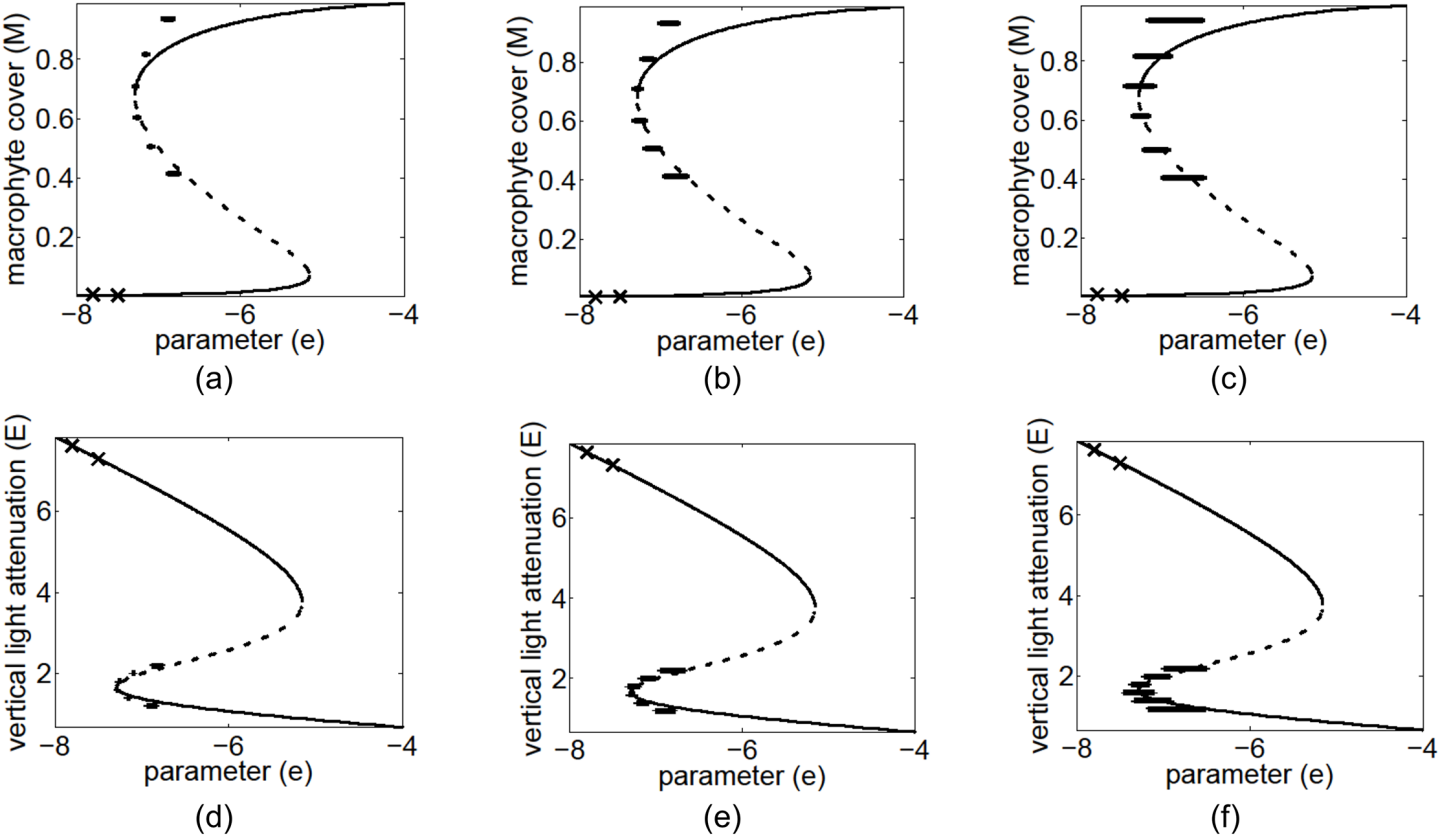
Predicted bifurcation diagrams for the feedback system between macrophytes and phytoplankton in a shallow lake using 100 recoveries at two pre-bifurcation conditions. The exact bifurcation diagram with both the stable (solid line) and unstable (dashed line) branches are shown in each plot. Transient data were collected at two locations (indicated by labels X) in the pre-bifurcation regime. The predicted post-bifurcation regime is shown by the standard deviation error bars for 100 separate noisy realizations for macrophyte cover with (a) 10% noise, (b) 20% noise, and (c) 30% noise, and vertical light attenuation with (d) 10% noise, (e) 20% noise, (f) 30% noise.

The analysis ultimately forecasts the value of the critical parameter for the nearest bifurcation, together with the states of the system after this transition (i.e. the bifurcation diagram). Results for the vegetation-grazing ecosystem are shown in Fig 6, and those for the feedback system of macrophytes and phytoplankton in a lake in Fig 7. In both these cases the predicted bifurcation marked the beginning of the bi-stable region (point *A* in Fig 2) Averages and standard deviation error bars are plotted for 100 separate noisy realizations. For the vegetation grazing ecosystem, 10%, 20% and 50% relative errors were added to the observed data, and 100 noisy recoveries were collected at each value of *c*. For the macrophytes and phytoplankton model, 10%, 20% and 30% relative errors were added to the observed data, and 100 noisy recoveries were collected at each value of *e*. In general, only one recovery is needed at each of at least two separate parameter values for the method to work, however additional recoveries help reduce the effects of noise.

The number of observations in the transient regime, along the recovery trajectory, range from about 20 (when farthest from the bifurcation) to 30 (when closest to the bifurcation) for the results shown in Fig 6. Similarly, the number of observations range from about 30 to 50 for the results shown in Fig 7. As the bifurcation is approached, more observations are available because data is collected at equal-time intervals, and the recovery rate slows down. The amount of data collected can be changed by increasing or decreasing the time between samples as the bifurcation is approached.

**Fig 8:**
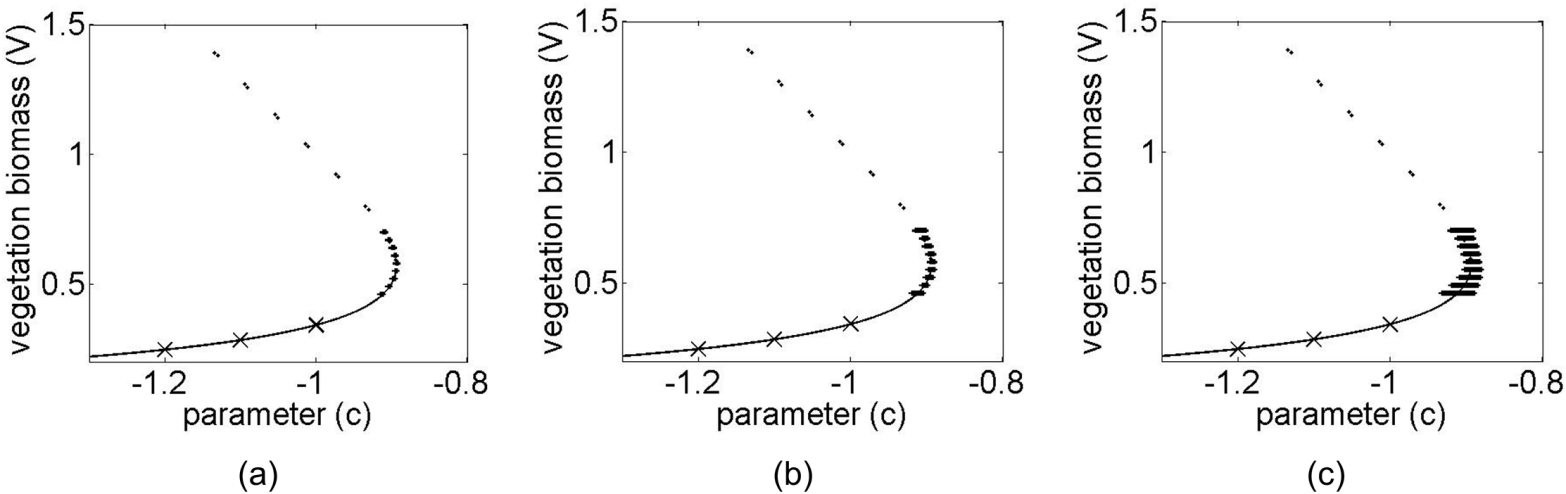
Predicted bifurcation diagrams for the vegetation model using a single recovery at three pre-bifurcation conditions. The exact bifurcation diagram with both the stable (solid line) and unstable (dashed line) branches are shown in each plot. Transient data were collected at three locations (indicated by labels X) in the pre-bifurcation regime. The predicted post-bifurcation regime is shown together with the standard deviation error bars for 100 separate noisy realizations for (a) 2% noise, (b) 5% noise, and (c) 10% noise.

Results from applying the method within the bi-stability region are shown in Fig 8 for the vegetation grazing ecosystem. These results show that the method can effectively be applied within the bi-stable region to predict the bifurcation. Noise levels of 2%, 5% and 10% were applied to the observed data, and *a single noisy recovery* was collected at each value of *c*. The number of data points measured in this case along the recovery trajectory ranged from 500 (when farthest from the bifurcation) to 800 (when closest to the bifurcation). The increased number of data points were needed in this case for noise filtering since only a single recovery was used. Therefore, for the case where only a single trajectory is sampled, the data must be sampled more frequently relative to the recovery rate of the system to its equilibrium point so that enough sampling points are obtained.

## Discussion

The approach proposed here takes advantage of observations of transient recoveries from large perturbations to forecast bifurcations together with the behavior of the system after these bifurcations. Large means here that the perturbations are not constrained to remain close to the initial, unperturbed, state of the system. On the contrary, excursions away from an initial equilibrium are relied on to obtain information about the state space. Predictions can be obtained without an explicit model, from either outside or inside the bi-stable region, especially when a potential jump to another equilibrium does not have undesirable consequences. The minimum requirement is measurement of transient trajectories from at least two parameter values.

This approach differs from the previous model-less method of Livina and Lenton [35] which is focused on analyzing many small perturbations, and the model-less method of Lade and Gross [36] which uses a generalized model and requires limited knowledge of structural information for the system. Those methods provide valuable qualitative information about an approaching bifurcation, and are often used after the system is already in the bi-stable region, but they give no information about the post-bifurcation regime.

Our results demonstrate the importance of exploiting transient recoveries of ecological systems while they recover from large perturbations. Ultimately, observing a system in its transient behavior tells much more about its dynamics and stability than observing its steady state properties. Van Geest et al. [23] have shown that transients can be critical in understanding the stability of a system when its dynamics are in between two stable states. In particular, they examined transient dynamics when two potential wells exist. This differs from the work presented here in that future dynamics involving bi-stability can be forecasted from transients in the parameter region where the system has only one stable state.

Measurements of the response of the system to large perturbations are used to estimate the value of *η* as a function of *x*. This function is a quantitative indicator of how the recovery rate of the system varies as it returns to its previous stable state. The recovery slows down the most near the bifurcation (e.g., indicated by points *A*, *B* and *C* in Fig 5(a)), which in turn corresponds to the largest values of *η* (e.g., indicated by points *A*, *B* and *C* in Fig 5(b)). The method uses the actual response of the system with no linearization in the state space.

The proposed method builds on the work of Lim and Epureanu [32, 33] which focused on electro-mechanical systems such as atomic force microscopes. That approach is expanded here to a broader class of nonlinear systems where very little is assumed about their dynamics. In the previous work, a variable $\lambda =\frac{\dot{x}}{x}$ was used, which limited the approach to a smaller class of bifurcations and required more knowledge of the system’s model. In the current work, the variable *η* is introduced which opens up the approach to a broader class of bifurcations including those of the two models studied in this work. In fact, the previous approach does not work properly for the two models studied here because it cannot handle S-shape bifurcations. In addition to broadening the scope of the bifurcations that can be handled, the best variable to measure to predict the bifurcation is considered in this work. Also, noise filtering techniques were examined and implemented to improve robustness to measurement noise.

Additional types of bifurcations such as transcritical bifurcations can be forecasted using this new approach. For these and other types of bifurcations the method would be carried out as described in the Methods section, however the plots in Fig 3 would have different qualitative features. For instance, for a transcritical bifurcation, there is no ghost node, but there is a critical slowing down in the recovery to the fixed point as the bifurcation is approached. Also, the *η* curve will have a different shape, instead of having the curved shapes shown in Fig 3, *η* would be a straight line.

Practical considerations follow on how the method can be applied to arbitrary systems with no knowledge on an underlying process-based model, on the identity of the bifurcation parameter, or even on whether there is a bifurcation. As expected, the more data are collected (at more parameter values or at higher sampling rates for each recovery, or repeatedly capturing data at the same conditions), the more accurate the forecasts will be. The robustness of the method to noise also improves as the bifurcation is approached. If the system is very far from the bifurcation, the effects of the critical slowing down will lessen. This would be apparent if the approach were used when the system is too far from criticality because then *η* would exhibit random values as opposed to the characteristic defined curvature illustrated in Fig 5(b). Random values of *η* would therefore indicate that the system is sufficiently far from a bifurcation, so that the method cannot detect it. In practice, the user would apply the method at whatever parameter is available. As the bifurcation is approached, the method becomes increasingly more accurate. When the *η* variable appears random, then either the system does not meet the assumptions or it is far from the bifurcation. As the parameter changes, new data would be obtained (for example in a monitoring approach). The *η* variable calculated with the new data will no longer be random when the system is close enough to the bifurcation for the method to detect the location of the bifurcation. When the bifurcation is close enough, this technique can be used to determine the type of bifurcation (subcritical vs. supercritical), the location of the bifurcation, and the states of the system after this bifurcation is crossed.

To apply the method to a system, one would first collect time series of the measured variable at multiple parameter values. These are the key inputs to the method. This information could be used to apply the method and examine the estimates obtained. If the system does not meet the necessary conditions, and/or if a poor choice of measurement variable is made, and/or a parameter that does not relate to the bifurcation parameter is used, then the *η* variable will not show a trend, but rather appear random (and the method will not work). This would be an indication that for that parameter, the system is not approaching a bifurcation (yet). The results would not guarantee that this would not happen as the parameter changes further, as we have no way to evaluate a priori the distance from the bifurcation at which the warning would be evident.

If a properly validated and calibrated model were available, then of course, one would not need to use the proposed method. However, such models are rarely available. Instead rough models may predict trends or indicate qualitative features of the dynamics without providing accurate quantitative forecasts. In such cases, these models can be used to ensure the assumptions of the proposed method are met, whereas application of the method itself can provide quantitative estimates of where the bifurcation will take place and what exactly the post-bifurcation regime will look like. In the best case scenario, of a properly validated and calibrated model that can provide accurate forecasts over a given time horizon, comparison of its predictions on the occurrence of a bifurcation to those by the proposed method would provide further validation of the model. Also, the other way around, this comparison would provide a useful demonstration of the method.

Additionally, there are some bifurcations for which this method is not well-suited, such as the period doubling one. In this case, the amplitudes of the dynamics in the pre- and post-bifurcation regimes are comparable. However, this type of bifurcation does not constitute a catastrophic transition.

Some knowledge of a quantity that relates to the bifurcation parameter is also needed. If one knows what the bifurcation parameter is, then this method can quantitatively predict when a bifurcation would occur. However, if one only knows a variable that relates to the bifurcation parameter, then the bifurcation cannot be identified precisely by this or any other method. Our method still provides however an estimate of where the bifurcation occurs as well as an estimate of the post-bifurcation dynamics. Essentially, information from Fig 5(b) could still be used qualitatively to gain understanding of the post bifurcation dynamics. Also, if the bifurcation parameter is completely unknown and/or recovery information is only available at a single parameter level, then the *η* curve can still be generated to gain insight into the post-bifurcation dynamics.

Additionally, for large dimensional systems, the selection of the proper variable to measure is important. A selection of a state most aligned with the inertial manifold is preferred (e.g., *E* in Fig 4). However, the shape of the manifold does not have to be known a priori. The selection can be made by exploring *η* (e.g., results in Fig 5(b)). A more curved *η* is obtained when the measured variable is along the inertial manifold. A flatter, horizontal line is obtained for *η* when the measured variable is nearly orthogonal to the inertial manifold.

When applied far from bifurcation, the method can use the higher-order terms (HOT) in Eq (1), namely, terms such as (*μ* − *μ*_*c*_)^2^, (*μ* − *μ*_*c*_)^3^ and so on. Inclusion of these terms will enhance the accuracy of the method further in advance of the bifurcation. The drawback is that their inclusion requires data collection at additional parameter values (an additional set of measured transient recoveries for each term included into Eq (1)). The optimal balance between prediction from farther away in parameter space and additional data requirements is of course application dependent. To determine whether HOT are needed (and if so, how high an order is needed) one can apply a classical convergence test. Specifically, one can include HOT (up to order *n*), collect sufficient data to account for these HOT, and then compare the predictions made without HOT and the predictions made with HOT (of order *n* and/or lower order such as *n* − 1 or lower). The differences among these predictions are indicative of the importance of the additional terms. In practice, this may be prohibitive for many system in nature, in which case initial application of the method and the qualitative results obtained, would indicate whether additional monitoring is important.

## Conclusions

The forecasting method presented here provides quantitative insights into a system behavior near and after bifurcations despite the lack of a process or statistical model. This is of relevance for ecological systems for which the construction and validation of accurate models is difficult. Although the accuracy of the forecasting increases when data are collected closer to the bifurcation, the method is applicable and accurate well in advance of the transition. The approach complements a recently proposed method that is also model-free and estimates the risk of both false alarms and failed detections in estimating tipping points, but applies specifically to saddle-node bifurcations and close to criticality [4]. Here, the dynamics of the system after the transition is also predicted, together with the type of bifurcation.

Note, that the four key assumptions needed for the method to work are not trivial, namely that (a) the parameter *μ* be close to *μ*_*c*_, (b) the inertial manifold be one dimensional, (c) the dynamics vary smoothly with the parameter, (d) the inertial manifold vary smoothly with the parameter. These assumptions hold for a variety of systems (such as the ones demonstrated) but are not universally true for all possible dynamics. A generic fingerprint of systems which obey all these assumptions (and especially assumption b) is not available, but is part of future work.

Future studies are underway to illustrate application to specific systems for which time series are available across natural and intervention-related perturbations. An open and promising area is that of taking advantage of management and intervention practices to examine the existence and location of bifurcations. In the case of infectious disease dynamics for example, interventions would be of interest to quantify how far one is from the elimination point, or in the opposite direction, from the point of emergence in the case of stuttering epidemics [37].

Further studies are also ongoing to determine the robustness of the method to system uncertainty (demographic and environmental process noise), to illustrate its application in seasonally-forced systems, and to detect how the prediction of the post-bifurcation dynamics changes as measurements approach the bifurcation. Also, optimal selection of measurement coordinates and their effect on robustness to noise are application-dependent and an important avenue of future work.
